# Identification of hot regions in protein-protein interactions by sequential pattern mining

**DOI:** 10.1186/1471-2105-8-S5-S8

**Published:** 2007-05-24

**Authors:** Chen-Ming Hsu, Chien-Yu Chen, Baw-Jhiune Liu, Chih-Chang Huang, Min-Hung Laio, Chien-Chieh Lin, Tzung-Lin Wu

**Affiliations:** 1 Department of Computer Science and Engineering, Yuan Ze University, Chung-Li, 320, Taiwan, R.O.C; 2 Department of Bio-industrial Mechatronics Engineering, National Taiwan University, Taipei, 106, Taiwan, R.O.C

## Abstract

**Background:**

Identification of protein interacting sites is an important task in computational molecular biology. As more and more protein sequences are deposited without available structural information, it is strongly desirable to predict protein binding regions by their sequences alone. This paper presents a pattern mining approach to tackle this problem. It is observed that a functional region of protein structures usually consists of several peptide segments linked with large wildcard regions. Thus, the proposed mining technology considers large irregular gaps when growing patterns, in order to find the residues that are simultaneously conserved but largely separated on the sequences. A derived pattern is called a cluster-like pattern since the discovered conserved residues are always grouped into several blocks, which each corresponds to a local conserved region on the protein sequence.

**Results:**

The experiments conducted in this work demonstrate that the derived long patterns automatically discover the important residues that form one or several hot regions of protein-protein interactions. The methodology is evaluated by conducting experiments on the web server MAGIIC-PRO based on a well known benchmark containing 220 protein chains from 72 distinct complexes. Among the tested 218 proteins, there are 900 sequential blocks discovered, 4.25 blocks per protein chain on average. About 92% of the derived blocks are observed to be clustered in space with at least one of the other blocks, and about 66% of the blocks are found to be near the interface of protein-protein interactions. It is summarized that for about 83% of the tested proteins, at least two interacting blocks can be discovered by this approach.

**Conclusion:**

This work aims to demonstrate that the important residues associated with the interface of protein-protein interactions may be automatically discovered by sequential pattern mining. The detected regions possess high conservation and thus are considered as the computational hot regions. This information would be useful to characterizing protein sequences, predicting protein function, finding potential partners, and facilitating protein docking for drug discovery.

## Background

Identification of functionally important regions directly from a protein sequence is a challenging problem in molecular biology [1-7]. Investigation of possible protein-protein interactions and prediction of the associated physical binding areas facilitate the study of all aspects of cellular function [8,9]. The principles that govern the interaction of two proteins and the general properties of their interacting interfaces remain uncovered [10-12], resulting in the difficulties of predicting interface regions directly from protein sequences. Even when the structure of a protein is available, it is still not a trivial task to localize the functional interfaces and to clarify the contribution of each involved residue [7,13,14].

Previous studies observed that not all the interface residues contribute the same level of free energy in a complex [15-17]. Using the alanine scanning mutagenesis [18], which estimates the energetic contribution of individual side-chains, it suggests that a small set of interface residues can contribute the most to the binding free energy [15,16,19]. These critical residues are called *hot spots*; they give rise to a significant increase in the absolute binding energy when mutated to alanine [15,16,20]. It is interestingly observed that hot spots are not uniformly spread along the interfaces. Instead, they are clustered as densely packed regions and are surrounded by energetically less important residues which might serve to occlude bulk solvent from the hot spots [15]. The assemblies of the hot spots and its neighboring moderately conserved residues are called *hot regions *[17]. A single or a few hot regions can be found in the interacting interface of two proteins [17,21]. Within the dense clusters, the hot spots and some moderately conserved residues both contribute to the stability of the complex [17].

Several approaches have attempted to predict interacting sites based on structure information [22-31]. Some of the approaches identify potential surface patches based on the shape of structures and then use features such as solvation potential, hydrophobicity, planarity, or accessible surface area to differentiate interacting sites from the other surface patches. Evolutionary information has also been demonstrated as a useful feature to this problem and widely employed when structures are available [32-36]. While little correlation between interface and conservation is observed at the level of amino acid side-chains [15,32,37-40], the conservation degrees of hot spots are more significant [15,17]. Several studies have shown that hot spots are usually more conserved than other surface residues and clustered in space [17,21,38]. It has been also shown that structurally conserved residues at protein-protein interfaces correlate with the experimental alanine-scanning hot spots [17]. In other words, the residues that affect the binding free energy dramatically tend to be strictly conserved during evolution. In this regard, Lichtarge *et al*. proposed an evolutionary trace method to facilitate the study of protein interfaces [13], followed by the development of an easy-to-use facility named ConSurf by Armon *et al*. in 2001 [7]. The procedure is based on extraction of functionally important residues from homologous proteins, and after that the conserved residues are mapped onto the protein surface to identify the functional interfaces [7,13].

The task becomes much more challenging when only sequence information is available. In such situation, the information about residue composition remains. Besides, evolutionary information is also available if there are sufficient homologues. In this regard, a classification scheme based on neural networks or support vector machines (SVM) with the features extracted from a sliding window on amino acid composition and evolutionary information is usually adopted [41-43]. Constructing a classifier requires a set of training data for which the protein structures are available. After that, the interacting residues of a query sequence can be predicted without structure information. Even though the information about which conserved residues form clusters in space is absent and cannot be exploited here, another observation from [42,44], interface residues tend to form clusters in sequence, has been aggressively employed in recent studies to refine the predicting results [41,42]. There also exist approaches that attempt to tackle this problem without learning from existing structures. Gallet *et al*. showed in their work that the interacting residues can be identified by hydrophobic moments [45].

As evolutionary information is demonstrated to be useful in finding interacting sites, we present here an alternative approach to discover conserved residues, sequential pattern mining [1,46,47]. Different from the evolutionary information derived by multiple sequence alignment of homologous sequences, the pattern mining approach focuses on the concurrence of several conserved blocks present in a subset of protein homologues [47]. Sequential pattern mining discovers a particular subsequence that frequently occurs among a set of sequences [46]. This technique has been widely used to identify protein motifs in many previous studies [48-50], where the term *motif *refers to such a subsequence that captures the characteristic regarding a specific biochemical function [51]. Finding functional motifs directly from protein sequences is challenging, because many sequence motifs are discontinuous and the spacing between motif elements is usually large and irregular [51]. By considering large flexible gaps in sequential pattern mining, the developed method can deliver long patterns spanning large wildcard regions efficiently [1,47]. Though the conserved blocks in our patterns are largely separated in sequences, they are often close to each other in 3D structures and play critical roles to protein functions [1]. The proposed methodology performs well even when the similarity identities between input sequences are low or the functional sites are only conserved in a few members of the input sequences [47,1]. This feature is important since it has been observed that residues that are conserved only in a specific subfamily may play more family-specific functional roles and are usually found at functional patches [5,6,14,52]. We expect that a highly supported pattern may highlight the residues that were conserved together during evolution for a particular purpose, for example, interacting with other proteins. The experimental results conducted in this work reveal that the conservation information provided by sequential pattern mining is helpful to this problem before any existing structures are included to facilitate the learning task.

This paper investigates the effectiveness of the approach by answering the following two questions: (1) are the locations of the sequential blocks near the interfaces of protein-protein interactions? and (2) do the derived sequential blocks tend to cluster together in space? Of course the first question is more related to the objective of this study. But by answering the second question, we expect to make it clearer why the proposed methodology works. We do not address the recall issue in this paper because we are aware of that it might not be possible to identify the complete set of interacting residues by a single pattern or in a single run of mining process. In fact, identifying important residues associated with hot regions is not identical to the problem of predicting interacting residues. As mentioned in the previous paragraphs, not all the interface residues are hot spots and expected to be conserved. On the other hand, some interior residues might also contribute to the stability of the complexes and are thus conserved. This work aims to show that the information provided by sequential pattern mining is useful to discovering hot regions of protein-protein interactions. This information can be refined and incorporated in other approaches to enhance the predicting power of the state of the art predictors.

## Results

In this section, we first describe the datasets used in this work and how the patterns are selected for different experiments. Using the five proteins in the first dataset, we investigate the potential of sequential pattern mining in identifying hot regions of protein-protein interactions by examining carefully the discovered patterns. To illustrate the advantages of our method, we compare our results with ConSurf's results. Next, we use the 220 protein chains of the second dataset to evaluate the general performance of the proposed method. The details of the datasets and the experimental procedures are described in the following subsections.

### Datasets

Table 1 lists the five proteins used in the first experiment. These proteins are selected randomly from available complexes in Protein Data Bank (PDB) [53]. For the second experiment, we collected 220 protein chains from the 72 protein complexes in the protein-protein docking benchmark 2.0 established by the ZDOCK team [54]. This benchmark contains protein complexes from several categories, including *enzyme-inhibitor*, *antigen-bound antibody*, *antibody-antigen*, and *others*, as summarized in Table 2. We further removed some similar protein chains from the second dataset by executing CD-HIT program [55] with 70% cut-off, resulting in a non-redundant dataset of the second dataset.

### Pattern selection

For the first dataset, the top ten large-size patterns are examined for the mining results of each query protein. The size of a pattern is defined as the number of conserved residues it contains. In the first experiment, it is observed in every case that the hot regions can be revealed directly by the maximum-size pattern. Thus in the second experiment, we investigate how the maximum-size pattern of each query protein performs in identifying protein interacting regions automatically.

### Results on the first dataset

The performance of the proposed methodology is evaluated from two aspects. First, the effectiveness of identifying hot regions is evaluated. Second, the efficiency of the pattern mining algorithm is compared with ConSurf, where multiple sequence alignment is employed in identifying conserved residues. In addition, the conservation plots generated by ConSurf are included for comparison.

**Figure 1:**
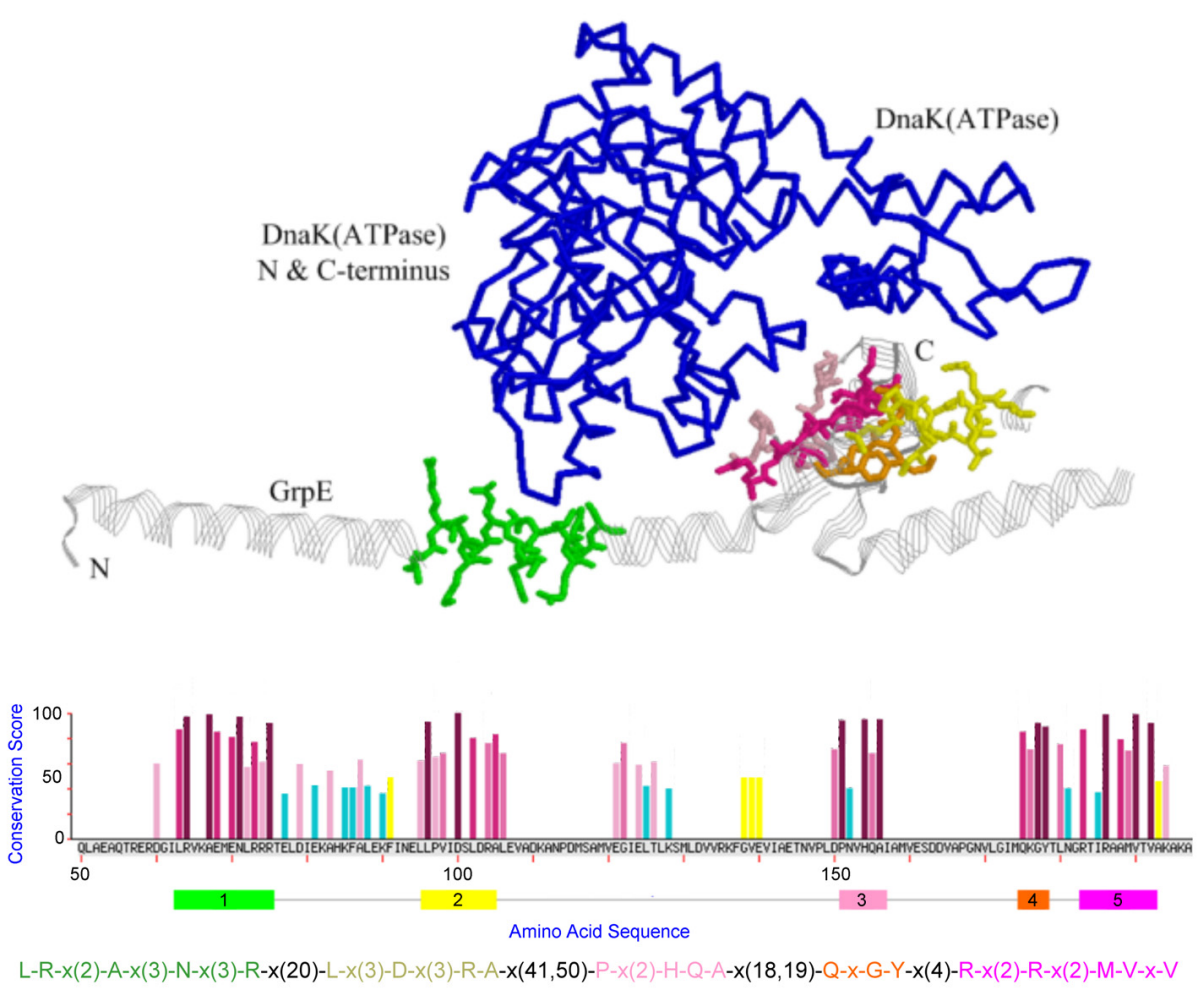
Representation of the GrpE-DnaKATPase complex [PDB:1dkg] with the pattern found by MAGIIC-PRO for GrpE protein. The pattern is plotted as *sticks*, GrpE as *strands*, and DnaKATPase as *backbone *display.

**Figure 2:**
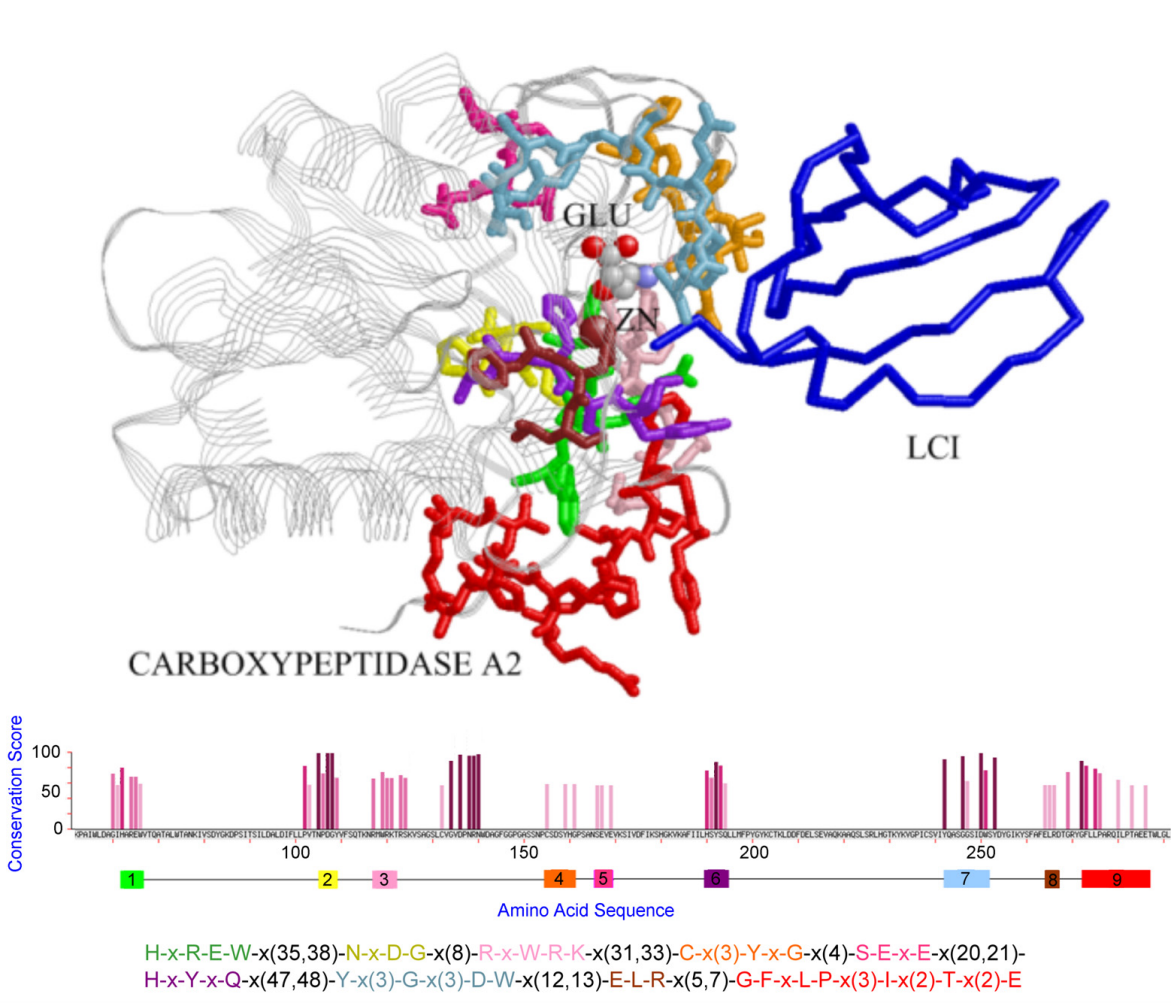
The pattern discovered for the PDB chain [PDB:1dtd, chain A], where the pattern blocks are shown in *sticks *with different blocks plotted in distinct colors, protein LCI in *backbone*, and zinc ions in crimson spheres. This maximum-size pattern hits the contact regions when interacting with the protein LCI, where the ligand GLU is plotted in *ball-and-stick *representation and colored in CPK mode.

**Figure 3:**
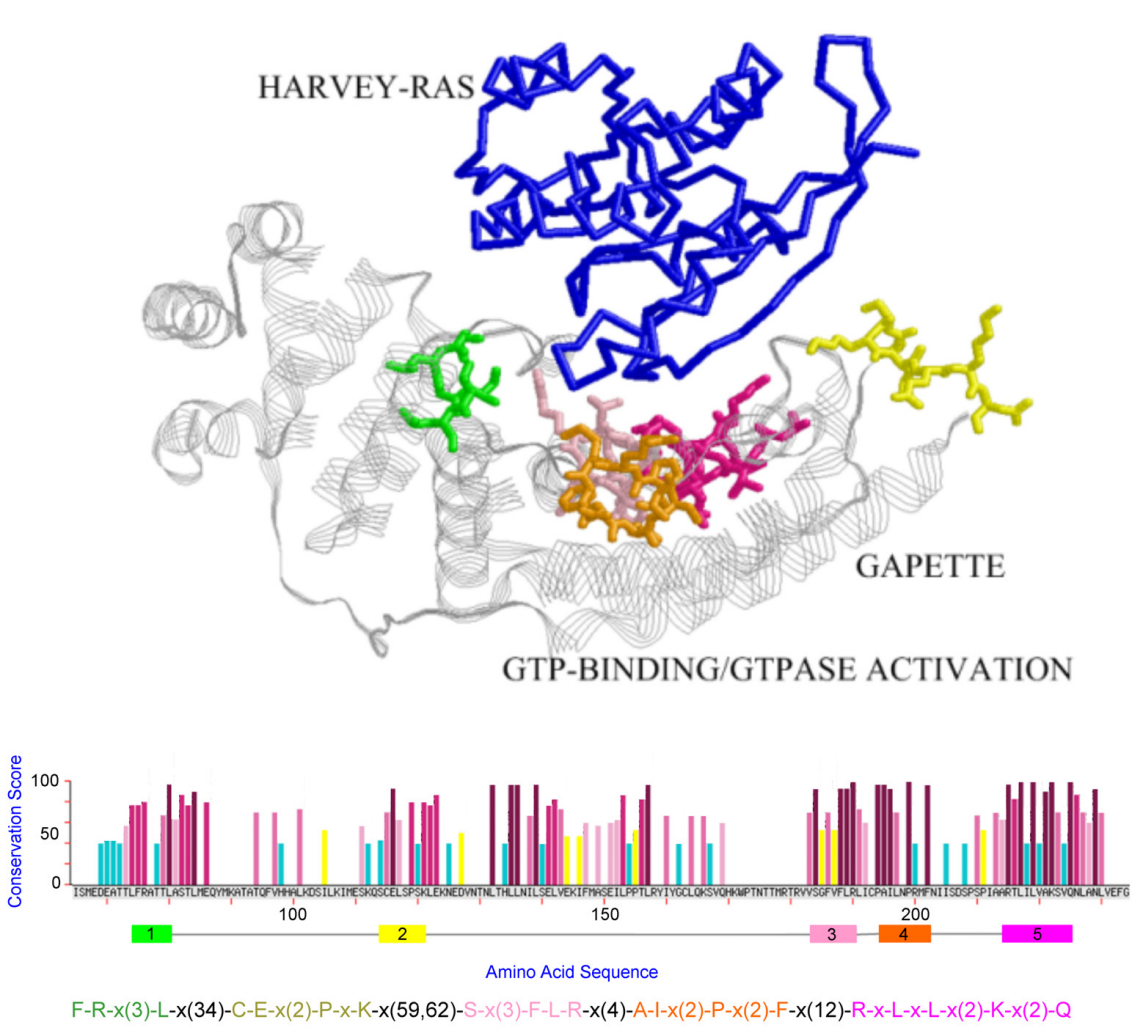
The pattern discovered for PDB chain [PDB:1wq1, chain G]. The pattern blocks are shown in *sticks *with different blocks plotted in distinct colors and HARVEY-RAS protein in *backbone*. The maximum-size pattern hits several contact regions of GAPETTE when interacting with the protein HARVEY-RAS.

**Figure 4:**
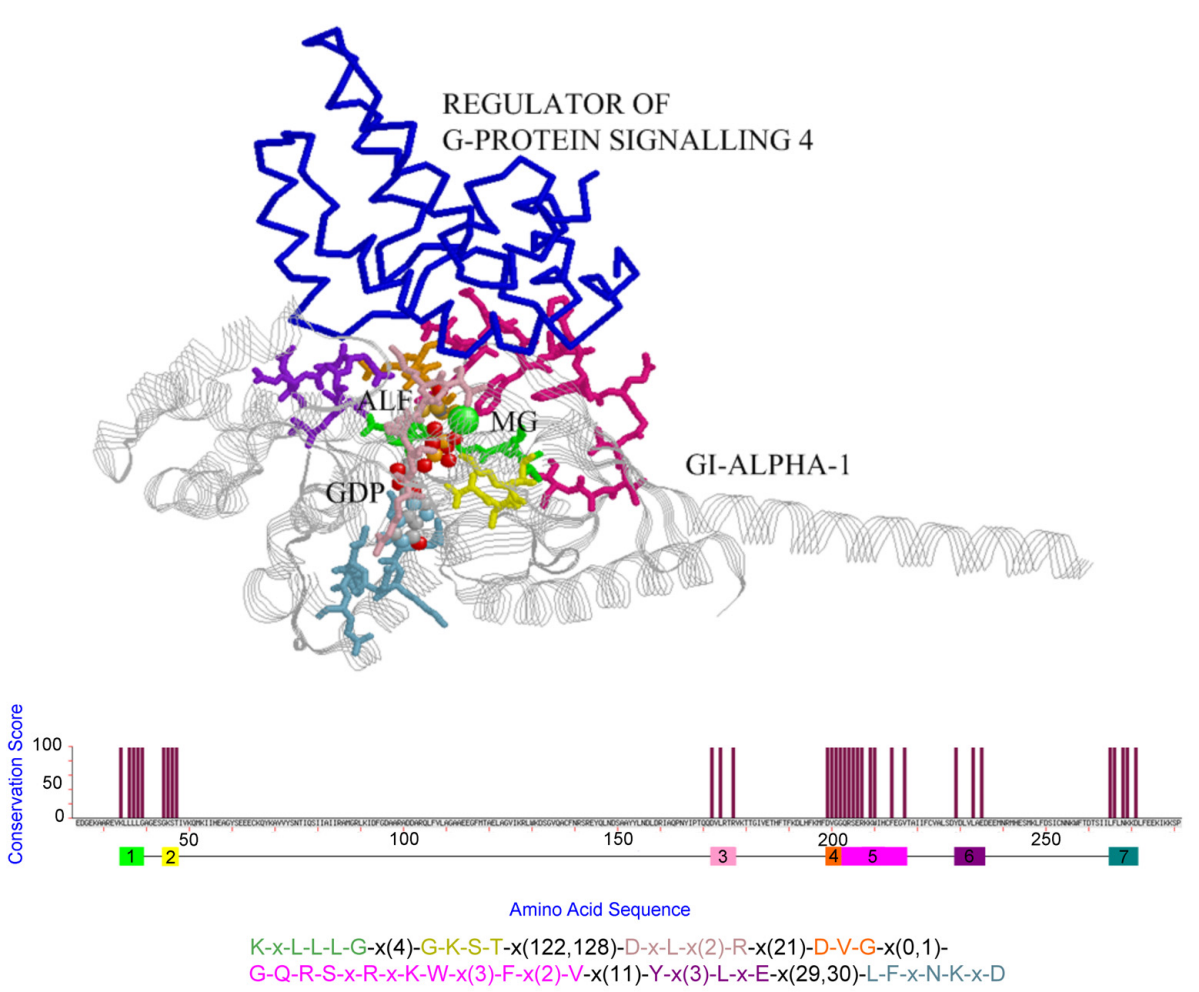
The pattern discovered for PDB chain [PDB:1agr, chain A], where the pattern blocks are shown in *sticks *with different blocks plotted in distinct colors and the regulator of G-protein signaling 4 is plotted in blue *backbone*. This maximum-size pattern hits the contact regions of GI-ALPHA-1 when interacting with the regulator of G-protein signaling 4.

**Figure 5:**
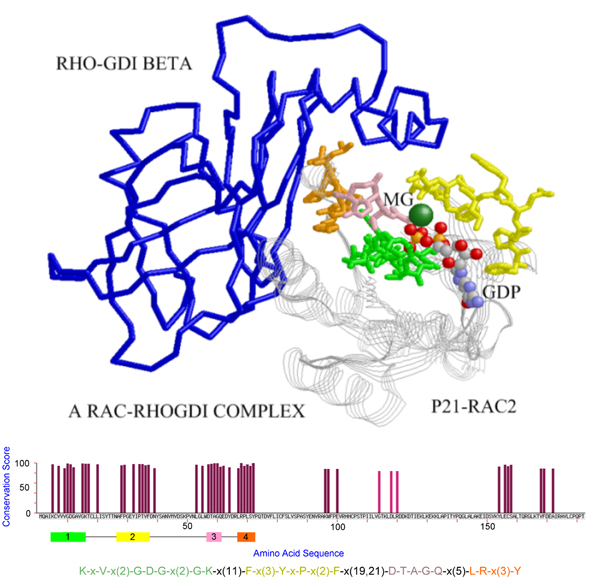
The pattern discovered for PDB chain [PDB:1ds6, chain A], where the pattern blocks are shown in *sticks *with different blocks plotted in distinct colors and the RHO-GDI Beta protein is plotted in blue *backbone*. This maximum-size pattern hits the contact regions of P21-RAC2 when interacting with the protein RHO-GDI Beta.

**Figure 6:**
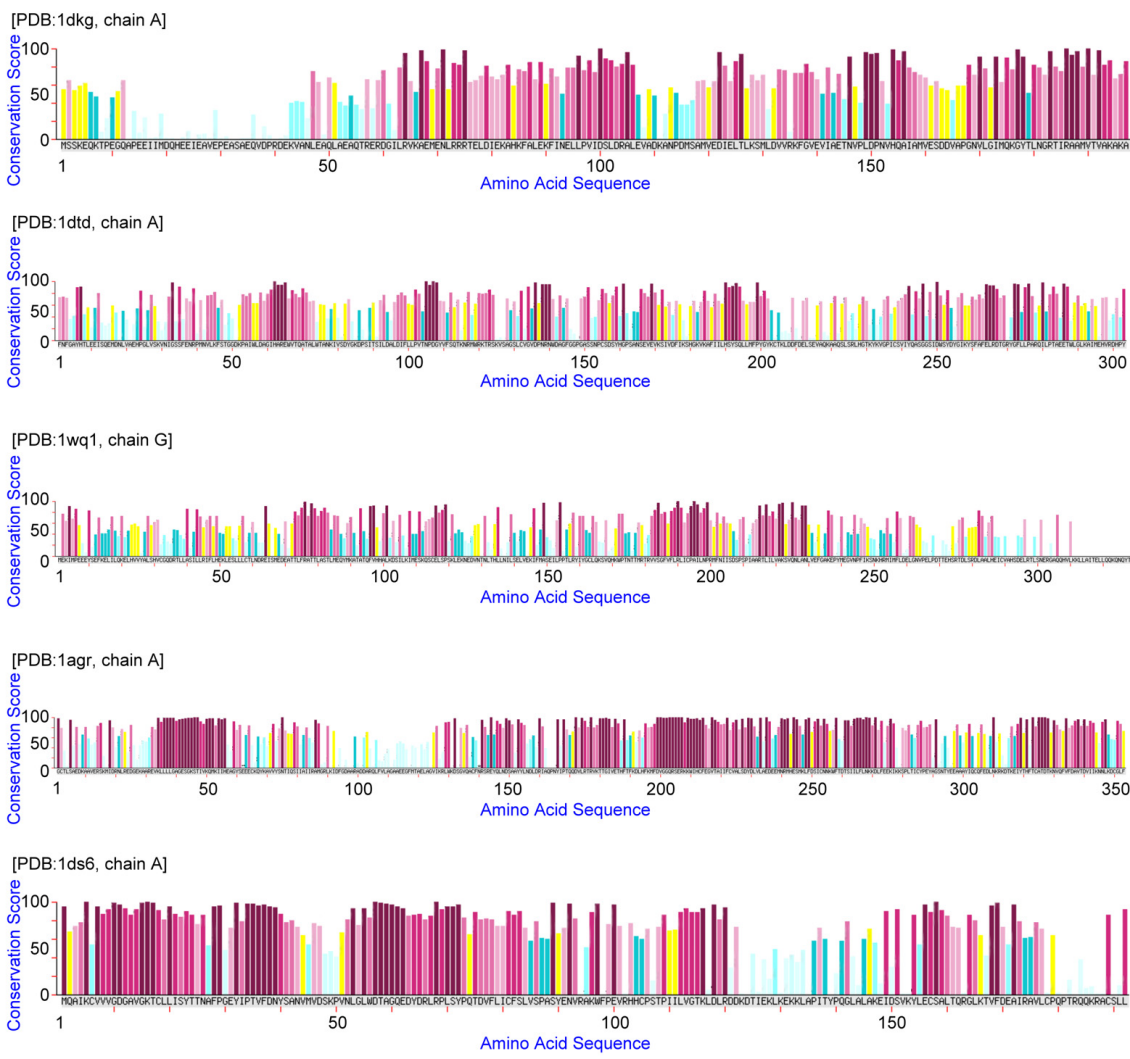
Conservation plots using the conservation scores calculated by ConSurf.

The mining results for the five proteins in the first dataset are shown in Figure 1, 2, 3, 4, 5, in that the patterns are plotted on the complexes for easy visualization. In these figures, the discovered conserved blocks are shown in *sticks *representation. In most cases, all the sequential blocks of the pattern cluster in one region of the protein and form the substructure associated with the interface of the complex. Differently, for the protein GrpE in Figure 1, the conserved blocks form two hot regions that together constitute the interacting interface. The conservation plots generated by MAGIIC-PRO are compared with that produced by ConSurf, as shown in Figure 6. The conservation information suggested by ConSurf might be too noisy to predict hot regions directly from the sequences. It would be helpful if the structures of complexes are available as suggested by Armon *et al*. and Lichtarge *et al*. in their papers [7,13]. Finally, Table 3 shows the executing time for MAGIIC-PRO and ConSurf respectively.

### Results on the second dataset

The summary of the experimental results on the second dataset is provided in Table 4, while the details can be found in the online supplement of this paper [56]. Among the 220 protein chains in the second dataset, two protein chains are excluded from the test set because the protein sequence of the protein chain [PDB:1ml0, chain B] is not available in the PDB file and the protein chain [PDB:1m10, chain A] does not have enough homologues for pattern mining (< 5 homologues). As listed in Table 4, MAGIIC-PRO successfully generated patterns for 212 protein chains. For each chain, we selected the pattern with the most components (called the maximum-size pattern) as the prediction of hot regions. Since only patterns with at least two blocks are reported, a maximum-size pattern always has two or more blocks to examine.

Here we define two indices to evaluate the quality of a pattern:

1. *Clustering propensity*: the percentage of sequential blocks in a pattern *P *that interacts with at least one of the other blocks in *P*. The interaction between a pair of blocks is defined by the following criterion: there exists an atom from one block that is within 5 Å to an atom of the other block.

2. *Interface propensity*: the percentage of sequential blocks in a pattern *P *that contacts another protein chain in the complex. The definition of contact is that any of the atoms from the block is within 7 Å to any atom of another protein chain in the complex.

The clustering propensity of a pattern reflects its reliability. We consider that a higher value of this index indicates that the pattern is more biologically meaningful, either from function or structure point of view. For each query protein, the clustering and interface propensities are calculated for its maximum-size pattern. The average values for different categories of protein complexes are provided in Table 5. The group of *enzyme-inhibitor *complexes slightly outperforms the other categories. It can be seen in Table 5 that the results on the non-redundant set are similar. When creating the non-redundant set, the program CD-HIT was applied directly to the 212 protein chains to avoid selecting the protein chains that failed to deliver patterns as the representatives.

Similar conclusion can be made from Table 4. As summarized in Table 4, there are about 66% of the derived blocks close to the contacting areas of protein-protein interactions. Furthermore, there are about 92% of the blocks clustering with at least one of the other blocks to form protein substructures in space. It is observed in some cases that some clustered but non-interacting blocks are actually the binding sites of other molecules (ligands).

In Table 6, we show the statistics about the number of blocks of the maximum-size patterns for the 218 protein chains. The number of blocks that contribute to interface is further collected in Table 7. In Table 7, it is of interest to check the number of proteins whose maximum-size pattern discovers at least two or three interacting blocks. The percentages are 83% and 54% respectively. We conclude that most of the tested proteins can be benefited by this approach, and similar records (80% and 51%) are observed on the non-redundant set in Table 8.

## Conclusion

Conservation information is important in predicting hot regions involved in protein-protein binding. However, the conservation information at residue level is not sufficient in predicting hot regions because not all the reported residues are conserved for the same purpose (the one studied in this paper is to preserve the environment of interacting with another protein). The conservation information derived by the pattern mining approach is more precise than that generated by multiple sequence alignment followed by constructing the evolutionary tree. That is, the concurrence of conserved blocks among a subset of protein homologues is focused. The experiments conducted in this paper reveal that the derived conserved blocks tend to cluster together in space and most of the aggregated blocks are related with interacting interfaces. The detected regions possess high conservation and thus are considered as the computational hot regions. By using sequential pattern mining, it may be possible to predict hot spots of an interface without exhaustive mutagenesis and thermodynamic analysis and thus the link between protein functions and their primary sequences can be constructed much more rapidly.

## Methods

In this section, we provide the details about the procedures of discovering and selecting patterns for predicting hot regions.

The residues associated with an interface are not necessarily found in one region of the sequence. Instead, it is usually observed that several remote segments of a protein sequence constitute a binding site [57-59]. Since it is time consuming to find long patterns with large irregular gaps, we recently presented a novel algorithm named MAGIIC to tackle this problem by using a combination of intra- and inter-block gap constraints [47]. In MAGIIC, the flexibility of intra-block gaps is limited, but the flexibility of inter-block gaps is largely relaxed. Using two types of gap constraints for different purposes improves the efficiency of mining process while keeping high accuracy of mining results.

The constraint model of MAGIIC has been refined in our recent work WildSpan [60] to enhance the capability of the mining algorithm in discovering functional motifs for a specific query protein. WildSpan restricts the length of intra-block gaps to be fixed, because it has been observed in previous studies that insertions and deletions are seldom present within highly conserved regions [17,59]. WildSpan further merges the upper and lower bounds of an inter-block gap into a single gap constraint called *maximum relative flexibility*. This constraint subsequently sets the upper and lower bounds of an inter-block gap with respect to the length of the gap observed on the query protein. The refinement of the constraint model reduces the complexity of the mining program and largely improves the accuracy of the derived patterns when functional motifs are desired. The idea of WildSpan was previously realized on the web server MAGIIC-PRO [1] to facilitate the whole process of discovering functional signatures from protein sequences. MAGIIC-PRO provides an easy-to-use environment in that the users can collect training data for a query protein by invoking PSI-BLAST [61] or Swiss-Prot [62] annotations. In addition, after the mining process completes, the derived patterns can be examined through several well-developed facilities [1].

A distinguishing characteristic of pattern mining from multiple sequence alignment in providing conservation information is that the residues in a pattern are simultaneously conserved among a certain amount of protein sequences in the training data. This property is appreciated from two points of view. First, a pattern collects a set of residues that are not necessarily the most highly conserved residues but are for sure to have been conserved simultaneously during evolution. Second, the pattern mining algorithm automatically identifies a subset of sequences from the training data that matches a particular pattern. Usually the resultant support rates are quite low, but it might still make sense since a sub-family could play family-specific functional roles [5,6,14,52]. We will explain more about why the concurrence of conserved residues in a set of protein sequences is important after introducing the concept of cluster-like patterns and the detailed mining procedures.

### Definition of cluster-like patterns and associated constraints

We call a pattern generated by WildSpan a cluster-like pattern. The residues inside a pattern are always clustered into several sequential blocks. The gaps in between two blocks are usually large and irregular. Here comes an example: "I-x-H-N-x(52,68)-E-x(2)-L-x-K-L". In this notation, a conserved residue is recorded by its amino acid symbol, 'x' denotes an arbitrary amino acid, x(*i*) stands for a gap of *i *arbitrary residues, and x(*i*, *j*), *i *<*j*, represents a wildcard region of at least *i *and at most *j *arbitrary residues. The shown pattern contains two conserved blocks "I-x-H-N" and "E-x(2)-L-x-K". The gaps within a block are called intra-block gaps, and the gaps in between two sequential blocks are called inter-block gaps. Concerning the efficiency of mining process, WildSpan specifies several constraints for these pattern components:

1. The maximum length of an intra-block gap: the length of intra-gap is rigid and cannot exceed the specified value.

2. The minimum number of residues in a block: a sequential block must contain at least a certain number of residues to eliminate noises.

3. The flexibility of an inter-block gap: a sequence can match a pattern as long as the inter-block gap does not violate the flexibility with respect to the query protein.

4. The minimum number of blocks in a pattern: a binding site is usually consisted of more than one protein segment. This constraint is set as 2 by default.

5. The minimum support of a pattern: the minimum percentage of sequences in the training data that match the derived pattern.

Setting minimum support is not an easy task. A loose bound may lead to explosion of patterns and cost a huge amount of computation, while a tight bound might result in no patterns. In MAGIIC-PRO, this issue is handled automatically by relaxing the minimum support constraint step by step until an expected number of desired patterns are discovered. In this regard, the patterns match the most input sequences will always be reported first.

### Description of WildSpan algorithm

Constraint-based sequential pattern mining extracts frequent patterns from unaligned sequences that satisfy the user-specified constraints, where pattern components maintain their order in the sequential data [63]. The algorithm WildSpan aims at discovering cluster-like patterns defined above by using a two-phase mining strategy. In the first phase, WildSpan generates the complete set of closed pattern blocks satisfying the block constraint and the intra-block gap constraint. A pattern or block is *closed *if none of its super-patterns getting exactly the same support (i.e. occurrence frequency). After that, in the second phase, WildSpan discovers the complete set of closed long patterns satisfying the inter-block gap constraint by connecting frequent blocks found in the first phase with large irregular gaps. Both the first and second phases execute a procedure call named *bounded-prefix-growth*, which was developed based on the function *prefix-growth *of a well known sequential pattern mining algorithm, PrefixSpan [46]. The *bounded-prefix-growth *procedure takes our new constraint framework into account, in order to match both the effectiveness and efficiency considerations. It uses a number of pruning strategies during the mining process. First, it exploits some good properties of the constraints to filter out many unpromising patterns/candidates in the early mining stage aggressively. Second, it recursively projects a sequence database into a smaller search space and grows patterns only in each projected database. Both features contribute to favorable mining efficiency. At the end of the second phase, WildSpan outputs the complete set of patterns that satisfy all the constraints specified by the users. The readers can refer to [60] for the details of the algorithm and [1] for the web server MAGIIC-PRO.

### Mining procedures

The complete procedures for identifying interacting interfaces for a query protein are as follows:

1. Obtaining homologues of a query protein (150 at most): This is achieved by running PSI-BLAST [61] against Swiss-Prot database [62] posted on Aug 4, 2005 with BLOSUM62 [64] substitution matrix and an *E*-value cut-off of 0.01. If the homologues of query protein are not sufficient in Swiss-Prot database (< 5 homologues), the searching is executed one more time against the non-redundant (NR) database [65] posted on Aug 4, 2005. The sequences nearly identical to the query protein (sequence identity from BLAST > 90%) or with a low identity (sequence identity from BLAST < 30%) against the query protein are further excluded from the training data.

2. Executing pattern mining: The minimum support is initially set as 100% and decreased repeatedly until at least one pattern with five blocks is discovered. A sequential block must contain as least three conserved residues, and the maximum length of an intra-block gap is 3. The mining process is terminated once the mining period exceeds four minutes in a single run, which often happens when the setting of minimum support constraint is too low such that the number of patterns explodes. If no patterns with five blocks can be reported with the previous settings, MAGIIC-PRO is invoked iteratively with the constraint on minimum number of blocks relaxed by one at a time.

3. Emerging information from all the patterns with two or more blocks into one conservation plot: The derived patterns are collected together to create a conservation plot. The conservation plot provides a whole picture about the conserved residues of a query protein. In this plot, the *conservation scores *are represented in different colors. The color level of a residue *x *is defined as: *L*(*x*) = *ceil*(9 × *R*(*x*)), where the conservation score *R*(*x*) is calculated by the following equation:

R(x)=conservation level of xmaximum conservation level among all the residues     (1)
 MathType@MTEF@5@5@+=feaafiart1ev1aaatCvAUfKttLearuWrP9MDH5MBPbIqV92AaeXatLxBI9gBaebbnrfifHhDYfgasaacH8akY=wiFfYdH8Gipec8Eeeu0xXdbba9frFj0=OqFfea0dXdd9vqai=hGuQ8kuc9pgc9s8qqaq=dirpe0xb9q8qiLsFr0=vr0=vr0dc8meaabaqaciaacaGaaeqabaqabeGadaaakeaacqWGsbGucqGGOaakcqWG4baEcqGGPaqkcqGH9aqpdaWcaaqaaiabbogaJjabb+gaVjabb6gaUjabbohaZjabbwgaLjabbkhaYjabbAha2jabbggaHjabbsha0jabbMgaPjabb+gaVjabb6gaUjabbccaGiabbYgaSjabbwgaLjabbAha2jabbwgaLjabbYgaSjabbccaGiabb+gaVjabbAgaMjabbccaGiabdIha4bqaaiabb2gaTjabbggaHjabbIha4jabbMgaPjabb2gaTjabbwha1jabb2gaTjabbccaGiabbogaJjabb+gaVjabb6gaUjabbohaZjabbwgaLjabbkhaYjabbAha2jabbggaHjabbsha0jabbMgaPjabb+gaVjabb6gaUjabbccaGiabbYgaSjabbwgaLjabbAha2jabbwgaLjabbYgaSjabbccaGiabbggaHjabb2gaTjabb+gaVjabb6gaUjabbEgaNjabbccaGiabbggaHjabbYgaSjabbYgaSjabbccaGiabbsha0jabbIgaOjabbwgaLjabbccaGiabbkhaYjabbwgaLjabbohaZjabbMgaPjabbsgaKjabbwha1jabbwgaLjabbohaZbaacaWLjaGaaCzcamaabmaabaGaeGymaedacaGLOaGaayzkaaaaaa@9353@

Here, the *conservation level *of each residue is determined by the percentage of total number of supporting proteins merged from different patterns.

The conservation plot is reported with the derived patterns to provide more detailed information when a pattern is examined.

**Figure 7:**
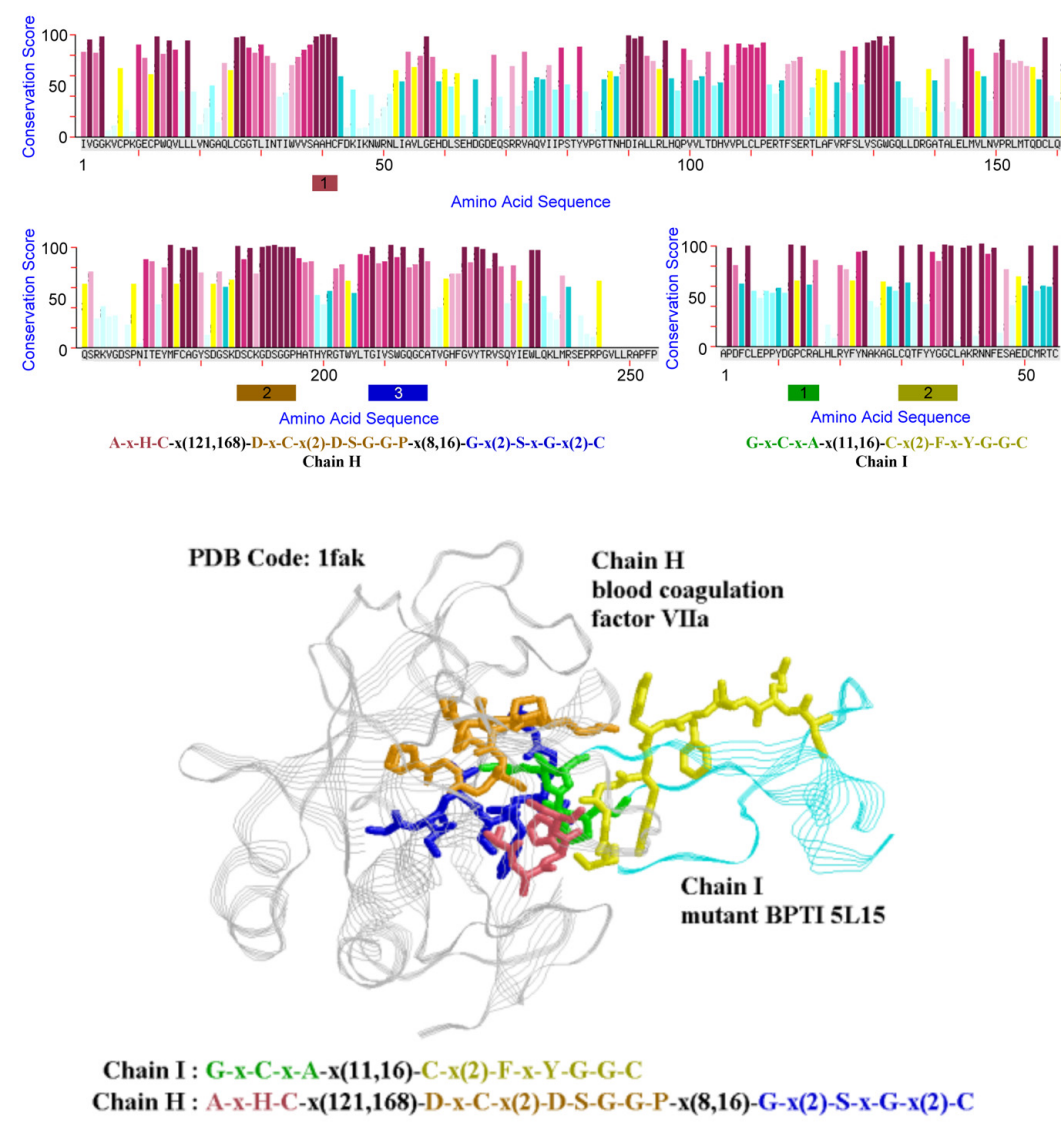
Example used to illustrate how the patterns generated by MAGIIC-PRO facilitate the study of identifying hot regions. The protruding residue Arg15 of 5L15 (chain I) falls in the first block of the derived pattern and the structurally conserved residues in the complemented pocket of VIIa (chain H) can be found in the three blocks of the derived pattern. The patterns are plotted as *sticks *representation on the structure and colored in the same way as in their regular expression form.

Here we use an example to illustrate the value of a long pattern when compared to traditional conservation scores. Figure 7 shows the complex of blood coagulation factor VIIa and a mutant of bovine pancreatic trypsin inhibitor 5L15 (chains H and I of PDB structure 1fak) with the residues in our pattern blocks highlighted by *sticks *representation. The conservation plots are generated based on the conservation scores calculated by the ConSurf server [66]. In this example, our patterns successfully detected the protruding residue Arg15 of 5L15 and most of the residues in the complemented pocket of VIIa addressed in [20]. It can be seen that many other residues are estimated to have similar conservation scores by ConSurf but are not present in the hot regions of this interaction. In other words, the conservation information of each residue alone is not sufficient for predicting hot regions. It is the concurrent conservation among a subset of respective homologues that suggests these residues might be conserved together for a specific purpose.

## Competing interests

The authors declare that they have no competing interests. See funding sources in Acknowledgements.

## Authors' contributions

CMH designed, performed all calculations and analyses, and drafted the manuscript. CYC provided guidance on design of the methodology, interpretation of the data, and manuscript preparation. BJL aided in the guidance on the study and provided financial support. CCH, CCL, LIAO, and TLW participated in the experiments.

## Figures and Tables

**Table 1:** Summary of the first dataset

Query protein (Swiss-Prot AC number)	Protein name	PDB complex (PDB entry : chain)
P09372	Protein grpE	1dkg:A
P48052	Carboxypeptidase A2 precursor	1dtd:A
P20936	Ras GTPase-activating protein 1	1wql:G
P10824	Guanine nucleotide-binding protein G(i)	1agr:A
P15153	Ras-related C3 botulinum toxin substrate 2	1ds6:A

**Table 2:** Summary of the second dataset, the protein-protein docking benchmark 2.0

Complex category	Number of complexes	Number of chains
Enzyme-Inhibitor/Substrate	23	51
Antigen-bound Antibody	12	35
Antibody-Antigen	10	30
Others	39	104
Total in the dataset	72	220

**Table 3:** Comparing the efficiency of MAGIIC-PRO and ConSurf

Query protein (PDB Code:Chain ID)	MAGIIC-PRO (seconds)	ConSurf (seconds)
P09372 (1dkg:A)	10	590
P48052 (1dtd:A)	15	191
P20936 (1wql:G)	69	122
P10824 (1agr:A)	16	472
P15153 (1ds6:A)	7	303

**Table 4:** Summary of the experimental results for the second dataset

Number of tested protein chains	218
Number of patterns examined	212
Number of discovered blocks	900
Average number of blocks per protein chain	4.25
Average time used for each protein chain	11.76 seconds
Number of blocks that is near interface	592 (~66%)
Number of blocks that form clusters	832 (~92%)
The maximum support of the patterns	100%
The minimum support of the patterns	13%
Average support of the patterns	66%

**Table 5:** Clustering and interface propensities of the patterns derived for different categories of the proteins in the second dataset

Complex Category	Average clustering propensity		Average interface propensity	
	Original	Non-redundant	Original	Non-redundant
Enzyme-Inhibitor/Substrate	90.24%	87.54	79.24%	74.46
Antigen-bound Antibody	96.11%	93.69	66.31%	64.05
Antibody-Antigen	95.56%	92.22	57.72%	50.00
Others	92.05%	90.16	67.58%	65.53
Total average in the dataset	92.77%	89.98	68.63%	66.28

**Table 6:** The statistics on the block numbers of the derived patterns for 218 protein chains of the second dataset.

Number of blocks: *x*		None	2	3	4	5	6	7	9
Patterns with *x *blocks	Number	6	18	32	74	71	10	3	4
	Percentage %	3	8	15	34	32	5	1	2
Patterns with at least *x *blocks	Number	218	212	194	162	88	17	7	4
	Percentage %	100	97	89	74	40	8	3	2

**Table 7:** The statistics on the number of interacting blocks of the derived patterns for 218 protein chains of the second dataset.

Number of blocks: *x*		None	1	2	3	4	5	6
Patterns with *x *interface blocks	Number	13	25	63	59	30	24	4
	Percentage %	6	11	29	27	14	11	2
Patterns with at least *x *interface blocks	Number	218	205	180	117	58	28	4
	Percentage %	100	94	83	54	27	13	2

**Table 8:** The statistics on the number of interacting blocks of the derived patterns for 138 non-redundant protein chains of the second dataset

Number of blocks: *x*		None	1	2	3	4	5	6
Patterns with *x *interface blocks	Number	12	22	40	35	18	15	2
	Percentage %	9	16	29	25	13	11	1
Patterns with at least *x *interface blocks	Number	138	132	110	70	35	17	2
	Percentage %	100	96	80	51	25	12	1
